# Physiological and transcriptomic analyses provide preliminary insights into the autotoxicity of *Lilium brownii*

**DOI:** 10.3389/fpls.2024.1330061

**Published:** 2024-05-14

**Authors:** Shumin Zhong, Chuibao Guo, Lu Su, Han Jiang, Xue-er Wang, Li Shi, Xiaogang Li, Xiaolan Liao, Jin Xue

**Affiliations:** Hunan Provincial Key Laboratory for Biology and Control of Plant Diseases and Insect Pests, College of Plant Protection, Hunan Agricultural University, Changsha, China

**Keywords:** Lilium brownii, phenolic, autotoxicity, transcriptome, reactive oxygen species (ROS), phytohormone

## Abstract

*Lilium brownii* F. E. Brown ex Miellez var. viridulum Baker (Longya lily) is a variety of *Lilium brownii* F.E. Br. ex Miellez. We used HS-SPME and GC-MS to screened the tissues of *L. brownii* roots, stems, bulbs, and leaves and obtained 2,4-DTBP as an autotoxic substance for subsequent analysis. 2,4-DTBP was highly autotoxic in some treatment groups. Based on changes in physiological indicators, we carried out transcriptomic analysis to investigate the mechanisms of autotoxicity of substances on *L. brownii* and obtained 188,505 Unigenes. GO and KEGG enrichment analyses showed that *L. brownii* responded differently to different concentrations and treatment times of 2,4-DTBP. We observed significant changes in genes associated with ROS, phytohormones, and MAPK signaling cascades. 2,4-DTBP affects chloroplasts, the integrity of the respiratory electron transport chain, and ribosomes, causing *L. brownii* autotoxicity. Our findings provide a practical genomic resource for future research on *L. brownii* autotoxicity and evidence for the mechanism of action of autotoxic substances.

## 1 Introduction

*Lilium brownii* is a perennial bulbous herb from the family Liliaceae that is used as a traditional Chinese herb. Its bulbs are rich in sugars, proteins, minerals, alkaloids, steroidal saponins, and other active ingredients, making it a popular natural health product that combines medicine and food. *L. brownii* is an important cash crop in Shao yang, Hunan Province, with economic benefits that are three to ten times greater than those of other crops. The *L. brownii* was designated as a Geographical Indication product in 2009, but there were severe Continuous Cropping Obstacles (CCO) in its production. For successive cultivations of *L. brownii*, soil sampling revealed significant acidification, along with severe outbreaks of pests and diseases, for which chemical control was inefficient.

Problems related to CCO have been reported in other crops (Zeng et al., 2020; Huang et al., 2021; Ku et al., 2022; Wang et al., 2022; Yan et al., 2022). Plant autotoxicity is thought to be a key contributing factor to the development of CCO. Autotoxicity affects the soil environment, microorganisms, and the crop itself by inhibiting the normal physiological functioning of chronically affected crops. Autotoxicity also causes soil acidification, nutrient imbalances, reduced enzyme activity, and an increase in pathogenic microorganisms (Gao et al., 2019; Chen et al., 2020a; Li et al., 2021). These effects have resulted in significant reductions in crop yields, increased rates of plant diseases, and the need to frequently change the land on which crops are grown, causing significant problems for agricultural production.

Secondary metabolite production is associated with plant adaptation to the environment in response to external stressors (Li et al., 2019b; Wu et al., 2020; Chen et al., 2020b; Yu et al., 2021). For example, phycocyanin and lignin are secondary metabolites that can act as biochemical barriers against invading pathogens (Ahuja et al., 2012; Gallego-Giraldo et al., 2018). Secondary metabolites are also involved in signaling plant disease resistance responses (Erb and Kliebenstein, 2020) and suppressing Herbivore-Induced defense signaling (Li et al., 2019a). Autotoxicity occurs when secondary metabolites produced by the decomposition of leachate and residues from plant roots, stems, and leaves, or chemicals secreted by a plant inhibit the growth of the plant’s roots, decrease its activity, and inhibit the growth of crops of the same species or the following crop.

The most commonly reported autotoxic substances are phenols and terpenoids, such as benzoic acid (Wu et al., 2015; Xiang et al., 2022), ferulic acid (Chi et al., 2013), p-hydroxybenzoic acid (Wu et al., 2021), and saponins. Autotoxic substances primarily affect the plant cell membrane, chloroplasts, mitochondria, and other cell structures, causing vesicle, cell membrane, and organelle destruction and affecting cell membrane permeability. Autotoxic substances influence plant physiological processes such as hormone homeostasis, oxidative stress response, cellular respiration, and photosynthesis (Kim et al., 2019; Yang et al., 2020; Zhang et al., 2020; Adamakis et al., 2021).

Few studies have investigated autotoxic substances in *L. brownii*. In this study, we isolated and identified the autotoxic substances that causes Continuous Cropping Obstacles from the tissues of *L. brownii*. We analyzed the activity of autotoxic substances and their effects on the physiological biochemistry of *L. brownii.* We used transcriptome sequencing to investigate the molecular mechanisms of autotoxic substance-induced stress in *L. brownii*. Our findings provide a theoretical foundation for further research into the autotoxic effects of *L. brownii* and aid in managing its Continuous Cropping Obstacles.

## 2 Materials and methods

### 2.1 Collection, separation, identification, and screening of autotoxic substances

#### 2.1.1 Collection of *L. brownii* tissue

Samples of fresh, healthy Longya lilies were collected from plants grown at the plant protection base after one-year cultivation at Hunan Agricultural University (28°10′44″N 113°04′29″), Hunan Province, China. The roots, bulbs, stems, and leaves were cleaned and dried at 45°C, then ground and stored at 4°C in the refrigerator for analysis.

Tissue culture plants of *L. brownii* were propagated in our laboratory.

#### 2.1.2 Isolation and identification of autotoxic substances and screening

Headspace solid-phase microextraction was used to isolate autotoxic substances from *L. brownii* samples. The headspace vial was preheated with 0.2 g of ground *L. brownii* for 40 min at 80°C, samples were then extracted for 40 min at 80°C followed by desorption for 5 min.

An GCMS-QP2010 system(Shimadzu, Japan) was used to identify the autotoxic substances isolated from *L. brownii*. The temperature of the injector and ion source was 240°C and 200 °C, respectively. The ionization was carried out in the electron impact (EI) mode at 70 eV. The capillary column was 30.0m length, 0.25 mm i.d., and 0.25 um film thickness. An initial column temperature was 50°C, retained 4 min, then increased to 200°C at a rate of 6°C/min and held for 10 min, and finally increased to 240°C at the rate of 8°C/min and held for 15 min. Helium was used as a carrier gas at a constant flow of 1.0 mL/min. The MS data were acquired in full scan mode from m/z 45~500 with an acquisition frequency of 2.5 scans per second, according to the method of Deng et al (Deng et al., 2004).

Results were compared and analyzed in the NIST database.

### 2.2 Bioassay of 2,4-DTBP treatment on *Allium ascalonicum*

*Lilium brownii* is a perennial plant with a long annual growth cycle that makes observation difficult in the early stages of growth, *Allium ascalonicum*, was selected for the test seed.

Using the GC-MS results, 2,4-DTBP solutions were prepared at concentrations of 0 (CK), 0.05, 0.1, 0.2, 0.4 and 0.8 g/L. 5 mL of each concentration was added to Petri dishes lined with filter paper. Healthy, uniform-sized *Allium ascalonicum* seeds were soaked at 50°C for 30 min, then left to cool naturally in a bottle containing 50°C water for 2 h. The seeds were then soaked in 5% sodium hypochlorite for 5 min, rinsed three times with sterile water and dried. The *A. ascalonicum* seeds were then evenly distributed in the Petri dishes and incubated at 25°C for 12 h in an illuminating incubator. The germination rate was recorded after 4 d. Root length and stem length were measured after 5 d. Twenty seeds per treatment were used, with three replicates per treatment, according to the method of Wang et al (Wang et al., 2018).

*L.brownii* tissue culture plants were treated with 0 (CK), 0.2, and 0.8 g/L of 2,4-DTBP to observe the effects of autotoxic substances.

Data are expressed as means ± standard errors. Differences were analyzed using one-way analysis of variance (ANOVA) and least significant difference (LSD) in SPSS 22.0 for Windows (SPSS, Inc., Chicago, IL, USA). P < 0.05 was considered significant.

### 2.3 Physiological and biochemical measurements of *Lilium brownii* after 2,4-DTBP treatment

*L. brownii* bulbs were placed in a hydroculture solution containing 0 (CK), 0.05, 0.2, and 0.8 g/L of 2,4-DTBP and sampled at 9 h, 12 h, 24 h, 72 h, and 120 h, and the content of superoxide dismutase (SOD), peroxidase (POD) activity, and malondialdehyde (MDA) was measured. Superoxide dismutase activity was determined using the nitrogen blue tetrazolium (NBT) method, peroxidase (POD) activity using the guaiacol method, and malondialdehyde (MDA) content using the thiobarbituric acid method.

### 2.4 Transcriptome sequencing of Lilium brownii under 2,4-DTBP stress

#### 2.4.1 Plant sample preparation

We used 0 (CK), 0.05, and 0.8 g/L 2,4-DTBP solutions for the *L. brownii* hydroculture. *L. brownii* root samples were collected at 9h, 24h, and 120h, washed with sterile water, blotted dry with sterile filter paper, immediately frozen in liquid nitrogen, and stored at -80°C in the refrigerator.

#### 2.4.2 RNA extraction, library construction, and sequencing

Total RNA was extracted using a Trizol reagent kit (Invitrogen, Carlsbad, CA, USA) according to the manufacturer’s protocol. RNA quality was assessed on an Agilent 2100 Bioanalyzer (Agilent Technologies, Palo Alto, CA, USA) and checked using RNase-free agarose gel electrophoresis. Prokaryotic mRNA was enriched by removing rRNA by Ribo-ZeroTM Magnetic Kit (Epicentre, Madison, WI, USA). The ligation products were size selected by agarose gel electrophoresis, PCR amplified, and sequenced using Illumina novaseq 6000 by Gene Denovo Biotechnology Co. (Guangzhou, China).

#### 2.4.3 Unigene function annotation

DESeq2 software was used to analyze RNA differential expression between two different groups (and by edgeR between two samples). Genes with a false discovery rate (FDR) below 0.05 and an absolute fold change ≥2 were considered differentially expressed. DEGs were analyzed using gene ontology (GO) and Kyoto Encyclopedia of Genes and Genomes (KEGG) enrichment.

### 2.5 Validation of expression profiles by qPCR

To verify the reliability of the RNA-seq results, nine genes with differential expression in each period were randomly selected for qPCR validation. The RNA extracted earlier was reverse transcribed using an Evo M-MLV RT Premix for qPCR (Accurate Biotechnology [Hunan]). We designed qPCR primers for the selected genes using Primer 3.0 (https://bioinfo.ut.ee/primer3-0.4.0/), with EIF and BHLH serving as internal reference genes. For the qPCR experiments we used the SYBR^®^ Green Premix Pro Taq HS qPCR Kit (Accurate Biotechnology [Hunan]) in the Bio-Rad CFX96 system. Relative expression levels were calculated using 2-ΔΔCt and normalized by the average of the two internal reference gene expressions. Each treatment had three biological replicates.

### 2.6 Tissue culture seedlings of *L. brownii* reprocessing with 2,4-DTBP

To confirm the impact of 2,4-DTBP on the oxidative phosphorylation and hormones of *L. brownii* as the transcriptome described, we treated tissue culture seedlings of *L. brownii* by the same method as the treatment did for the transcriptome analysis. Tissue culture seedlings with consistent growth of *L. brownii* were sampled after treatment with 0, 0.2 and 0.8 g/L of 2,4-DTBP for 0, 9 and 120 hours, respectively. Seedlings were planted in rooting medium contenting 2.3 g/L MS, 0.1 mg/L NAA, 7 g/L agar and 25 g/L sucrose. The medium was supplemented with 2,4-DTBP mother liquor to a final concentration of 0, 0.2, and 0.8 g/L. Each treatment had three replicates. DEGs which are pertinent with oxidative phosphorylation and hormones pathways were chosen for validation. The succinate dehydrogenase (SDH) was extracted and the activity was determined by following the procedure outlined in the SDH test kit (Solarbio, BC0950).

## 3 Results

### 3.1 Isolation and identification of autotoxic substances and screening

**Figure 1:**
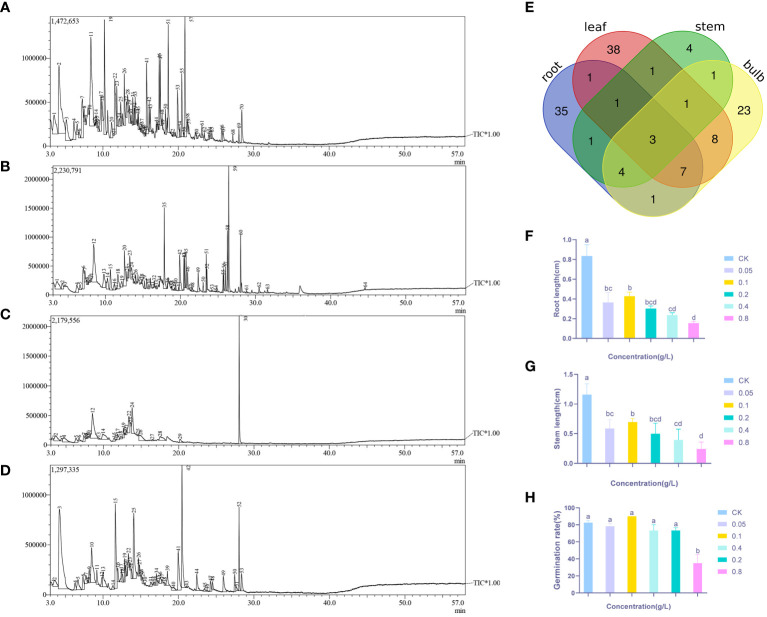
GC-MS of *L. brownii* four tissues, **(A)** Leaf **(B)** Root **(C)** Stem **(D)** Bulb, and **(E)** Venn diagram of GC-MS analysis results for the four tissue. Effects of different concentrations of 2,4-DTBP on **(F)** Root length **(G)** Stem length **(H)** Germination rate of Allium ascalonicum. Values are mean ± SEM. Different letters indicate statistical differences according to ANOVA (P < 0.05).

Headspace solid-phase microextraction followed by GC-MS (**Figures 1A–D**) was performed on the roots, bulbs, stems, and leaves of *Lilium brownii*. There were 64 peaks with more than 80% similarity. The compounds identified from the four tissues were categorized and showed a high degree of variability between the tissues (**Supplementary Table S1**). The roots, bulbs, and leaves contained the most compounds with 53, 48, and 60 compounds, respectively (**Table 1**). Only 16 compounds were isolated from the stems. The roots contained primarily phenol and alcohol compounds, while the leaves and bulbs contained mainly aldehydes. No ester compounds were identified in the stems. The roots, stems leaves, and bulbs contained heptadecane, 2,4-di-tert-butylphenol, and 2,4-dimethyl benzaldehyde, and all expect bulbs contained 4-methyl dodecane. The bulbs, roots, and stems contained n-hexadecane, 2-ethyl hexanol, (2-methyl propyl) nonane, and hexanal, while the leaves, bulbs, and stems contained n-tetradecane. The three most diverse compounds shared by bulbs, leaves, and roots were decanal, heptanoic acid, n-dodecane, D-(-)-pantolactone, octanoic acid, trans-2-noninal, and hexanoic acid.

**Table 1:** Results of GC-MS analysis of *L. brownii*.

substance	tissues			
	roots	stems	bulbs	leaves
phenols	11	1	1	1
aldehydes	4	2	12	14
esters	5	/	5	7
alcohols	16	2	7	4
organic acids	5	1	6	4
others	12	10	17	30
total	53	16	48	60

The majority of autotoxic substances were phenolic, ester, saponin, and other compounds. Screening 2,4-DTBP, an autotoxic substance, was performed by combining the number of occurrences of each compound in each tissue of the plant in the Venn diagram (**Figure 1E**). The effect of 2,4-DTBP on *Allium ascalonicum* (Liliaceae crop) root length, stem length, and seed germination rate was determined separately (**Figures 1F–H**).

### 3.2 Effect of 2,4-DTBP on the enzymatic activity of *Lilium brownii*

**Figure 2:**
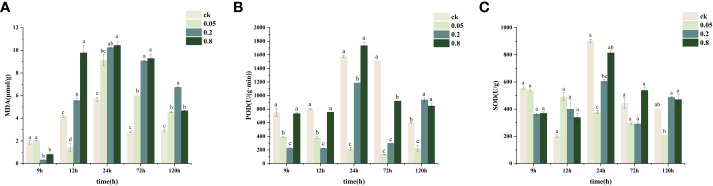
Changes in physiological indices of hydroponically grown Lilium brownii from 9h to 120h. **(A)** MDA content **(B)** Peroxidase activity **(C)** Superoxide dismutase activity Values are mean + SEM. All experiments were repeated at least three times with similar results.

POD and SOD activities in CK-treated *L. brownii* increased significantly after 24 h (**Figures 2B, C**). The high level of POD activity remained constant at 72 h, whereas the MDA content was elevated between 12-24 h but then leveled off between 24-72 h. POD and SOD activities were inhibited by 2,4-DTBP at concentrations of 0.05 g/L and 0.2 g/L compared to CK. At 0.2 g/L 2,4-DTBP, POD and SOD activities increased between 9h and 24 h reaching a peak at 120 h. In contrast, the MDA content remained the same. Additionally, the increase in POD activity did not inhibit membrane lipid peroxidation. Under 0.8 g/L 2,4-DTBP treatment conditions, POD and SOD enzyme activity remained at the same level as CK. In contrast, MDA content increased significantly compared to CK at the beginning of 12 h and remained at the same level as CK in the subsequent process. High concentrations of 2,4-DTBP treatment caused irreversible damage to the cell membrane structure of *L. brownii*, and changes in antioxidant enzyme activity could not eliminate the stressing effects caused by autotoxic substances.

The MDA content increased with an increase in 2,4-DTBP concentration (**Figure 2A**), reaching a peak at 24 h. Treatment with a high concentration of 2,4-DTBP accelerated the onset of lipid peroxidation in *L. brownii*. At 12 h, the MDA content in the treatment group increased with the concentration of 2,4-DTBP. The MDA content in all treatment groups returned to relatively low levels at 120 h but was still significantly higher than CK. To investigate the effect of 2,4-DTBP on *L. brownii* under different conditions, we treated roots with CK, 0.05 g/L, and 0.8 g/L 2,4-DTBP for 9h, 24h, and 120h, and collected samples for transcriptome sequencing.

### 3.3 Transcriptome analysis of *Lilium brownii*

**Figure 3:**
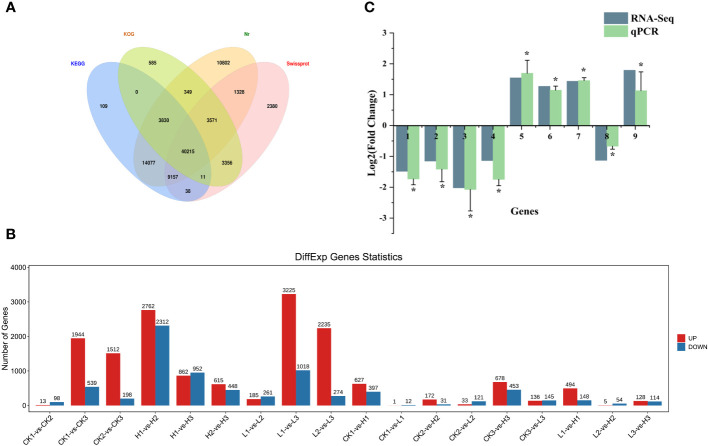
**(A)** Venn diagram of Unigene function annotation of Lilium brownii. **(B)** Statistical analysis of differentially expressed genes among samples of Lilium brownii. **(C)** Validation for the expression of selected DEGs by qPCR. The qPCR results were analyzed by an independent-sample t-test with a significance level of P<0.05.

The 27 samples sequenced generated raw data ranging from 37937896 to 63057410 Unigenes, with 99% clean reads and a Q20 greater than 98% (**Supplementary Table S2**). The clean reads were assembled using Trinity software 2.1.1, and a total of 188505 Unigenes were obtained with an average length of 807bp, an N50 of 1356bp, a maximum splicing length of 17880bp, and a minimum of 201bp. There were 67437 (35.77%), 51917 (27.54%), 83,329 (44.21%), and 60056 (31.86%) similar Unigene sequences annotated in KEGG, KOG, Nr, and Swiss-Prot, respectively. 40215 Unigenes were simultaneously annotated by four major databases, accounting for 21.33% of the total Unigenes (**Figure 3A**).

We used analysis of variance to screen significantly different genes with an FDR <0.05 and |log2FC|>1. Different pairs of groups were compared and the up- and downregulated genes were counted separately (**Figure 3B**). The number of differential genes varied among the different groups. The control and high-concentration treatment groups had the highest number of differential genes at 120h, with 678 upregulated and 453 downregulated. Enrichment analysis was performed after classification of differential genes using volcano and Wayne plots. KEGG pathway and GO annotations with an FDR < 0.05 were selected for discussion. QPCR was used to investigate the relative expression levels of nine randomly selected differential genes to validate the RNA sequencing results (**Figure 3C**).

### 3.4 DEG analysis of the effects 2,4-DTBP concentration gradient on *L. brownii*

**Figure 4:**
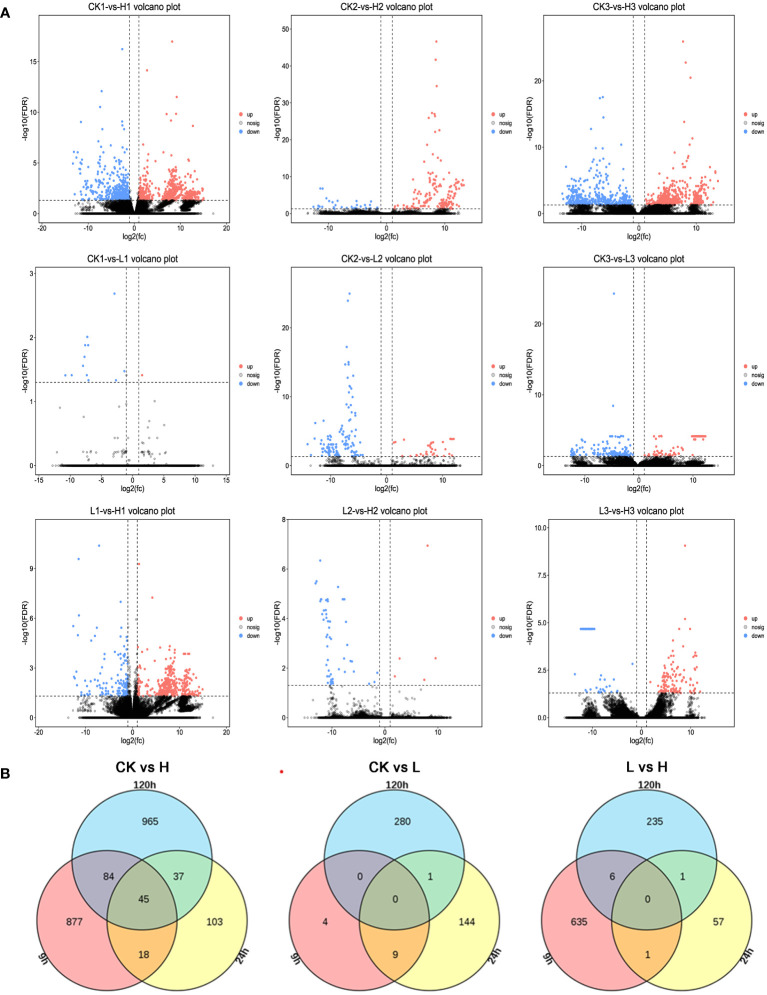
**(A)** Comparison of volcano plots with different concentrations at the same time. The number of DEGs in each tissue is presented and based on the significance shown in the volcano plots. The vertical dash lines in the volcano plots depict the two-fold differential expression cut-off (axis expressed as log2 values) and the horizontal dash lines shows the -log10(P-value). Dots in red (group_2 up-regulated relative to group_1) and blue (down-regulated) indicate differences in gene expression (FDR <0.05), while black dots show no differences. **(B)** Comparison of different treatment groups, Venn diagram of DEGs between various periods.

Groups treated with higher concentrations of 2,4-DTBP had more active gene expression differences, with a higher number of differential genes at 9h, 24h, and 120h than those treated with lower concentrations of 2,4-DTBP (**Figure 4A**). There were only four genes with significant difference in gene expression after 9 h of treatment with a low concentration of 2,4-DTBP compared to the control. However, after 24 h a significant change in the number of differential genes was observed.

**Figure 5:**
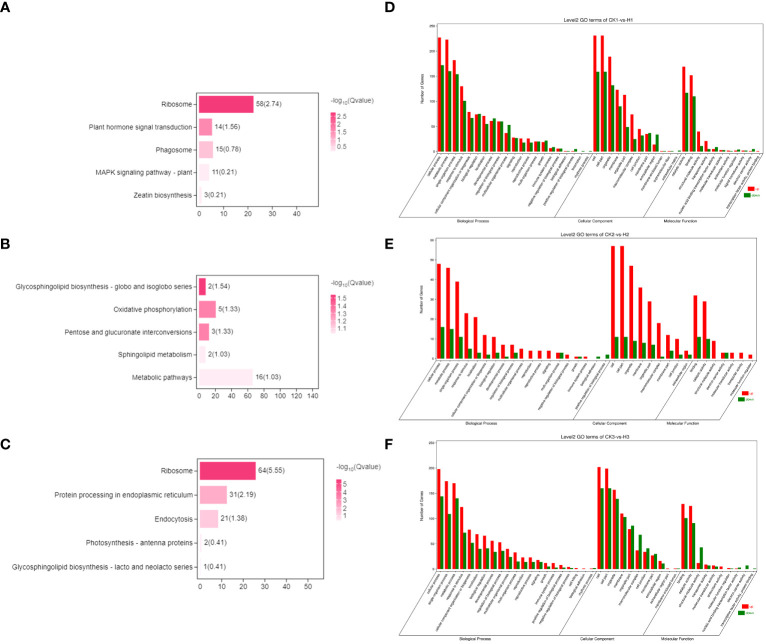
The enriched KEGG pathways of the DEGs. Choosing DEGs was only specifically present in various periods of the high-concentration treatment group. **(A)** 9h **(B)** 24h **(C)** 120h. The horizontal axis describes the name of the pathway, and the vertical axis shows the number of genes in the pathway. GO functional annotation of the DEGs. **(D)** 9h **(E)** 24h **(F)** 120h.

While the high concentration treatment and control groups shared a similar number of differential genes at 9h and 120h, less than 20% were identical (**Figure 4B**). The specific differential genes present in each of the three time periods were analyzed separately for enrichment analysis (**Figures 5A–C**). Genes identified at 9h were mainly hormone signaling and ribosome-related genes. GO analysis revealed that the associated differential genes were primarily related to cell wall formation and response to external influences. After 24h, differential genes were mainly associated with mitochondrial membranes and cellular respiration, and at 120h they were mostly associated with cell structure (**Figures 5D–F**).

**Figure 6:**
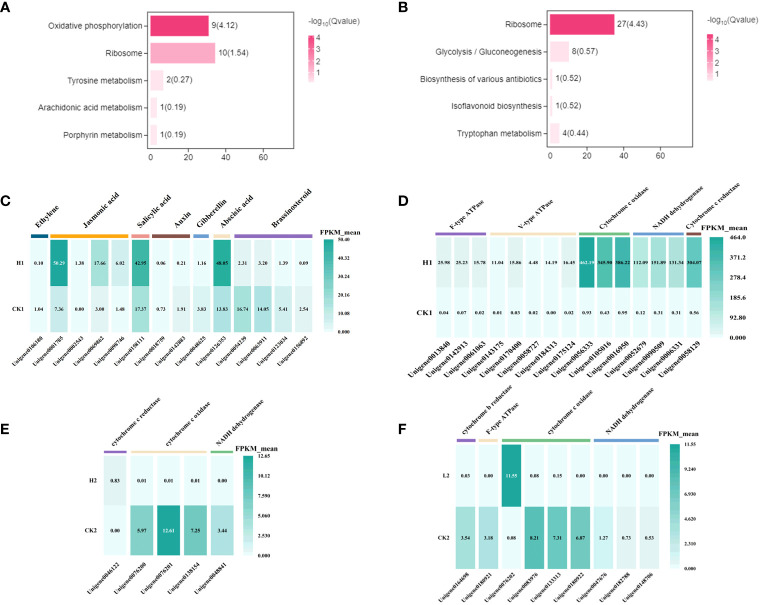
The enriched KEGG pathways of the DEGs. Choosing DEGs was only specifically present in various periods of the low-concentration treatment group. **(A)** 9h **(B)** 24h. The vertical axis describes the name of the pathway, and the horizontal axis shows the number of genes in the pathway. **(C)** Heatmap representing expression dynamics of genes involved in phytohormones at 9h CK vs H. group. **(D)** Heatmap representing expression dynamics of genes involved in Oxidative phosphorylation at 9h CK vs H. group. Heatmap representing expression dynamics of genes involved in Oxidative phosphorylation at 24h **(E)** CK vs H. and **(F)** CK vs L. group.

Overall, there was an increase in the number of specific differential genes in the low concentration 2,4-DTBP treatment group at 9h, 24h, and 120h compared to the control group, However, within the treatment group there were fewer differential genes at 9h compared to 24h and 120h. KEGG analysis revealed that 24h-specific differential genes were enriched in the Oxidative phosphorylation (Ko00190) and Ribosome (Ko03010) pathways, and at 120h differential genes were primarily enriched in the Ribosome (Ko03010) pathway (**Figures 6A, B**).

A stress response was observed in *L. brownii* after 9 h treatment with high concentrations of 2,4-DTBP. Auxin, gibberellin, ethylene, and brassinosteroid-related genes in the hormone signal transduction pathway (Ko04075) were significantly downregulated, while abscisic acid, jasmonic acid, and salicylic acid-related genes were significantly upregulated (**Figure 6C**). After 24 h, annotations related to respiration, mitochondrial membrane, and electron transport were enriched in both treatment groups compared to CK. However, oxidative phosphorylation-related mitochondrial complexes, cell wall, and endoplasmic reticulum-related genes were differentially expressed in the high-concentration treatment group but not in the low-concentration treatment group.

After 9h, oxidative phosphorylation-related genes were upregulated in the high-concentration treatment group (**Figure 6D**), but the level of upregulation varied by gene. Furthermore, the 9h annotated transcripts of genes for NADPH dehydrogenase, cytochrome C oxidase, and cytochrome C reductase were significantly more upregulated than for ATPase but not Unigene 0076202 (**Figures 6E, F**).

### 3.5 DEG analysis of the effects of 2,4-DTBP treatment time on *L. brownii*

**Figure 7:**
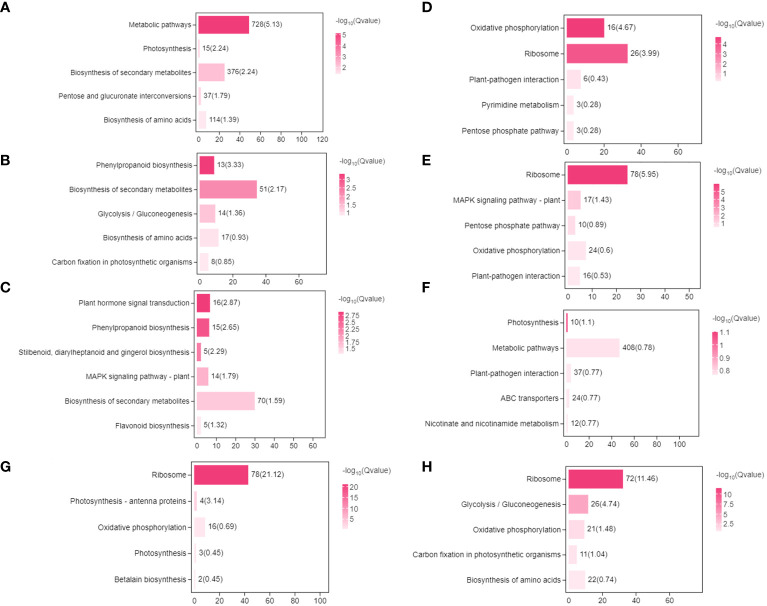
The enriched KEGG pathways of the DEGs. Choosing DEGs was only specifically present in various periods or treatment group. High-concentration treatment group **(A–C)**, **(A)** 9-24h **(B)** 24-120h **(C)** 9-120h. Low-concentration treatment group **(D–F)**, **(D)** 9-24h **(E)** 24-120h **(F)** 9-120h. Control group **(G, H)**, **(G)** 24-120h **(H)** 9-120h.

After 24h, the number of differential genes specific to the high-concentration treatment group were several-fold higher than in the low-concentration treatment group and CK, and differentially expressed genes were mainly enriched in primary and secondary metabolic pathways and photosynthesis (**Figure 7A**). GO enrichment analysis identified 77 biological process annotations that were significantly enriched in response to 2,4-DTBP treatment (**Supplementary Table S3**). These were mainly related to the photosynthetic membrane (GO:0034357), cell wall (GO:0005618), cytoplasm (GO:0005737), and other structures in the cell membrane (GO:0016020), all of which are associated with oxidoreductase activity (GO:0016491). Under low concentration 2,4-DTBP treatment conditions, only the oxidative phosphorylation (Ko00190) and ribosome (Ko03010) pathways were significantly enriched (**Figure 7D**), with the differential genes primarily involved in processes relating to cellular respiration, including the electron transport chain.

After 120h, the differential genes in CK were associated with cell structure (**Figure 7G**). Low-concentration treatment resulted in differential genes enriched in ribosomes (Ko03010) and MAPK signaling pathway-plant (Ko04016) (**Figure 7E**) which are involved in RNA modification (GO:0009451) and ribonucleoprotein complex biogenesis (GO:0022613) processes. High-concentration treatment for 120h enriched more secondary metabolic pathways than 24h treatment (**Figure 7B**).

**Figure 8:**
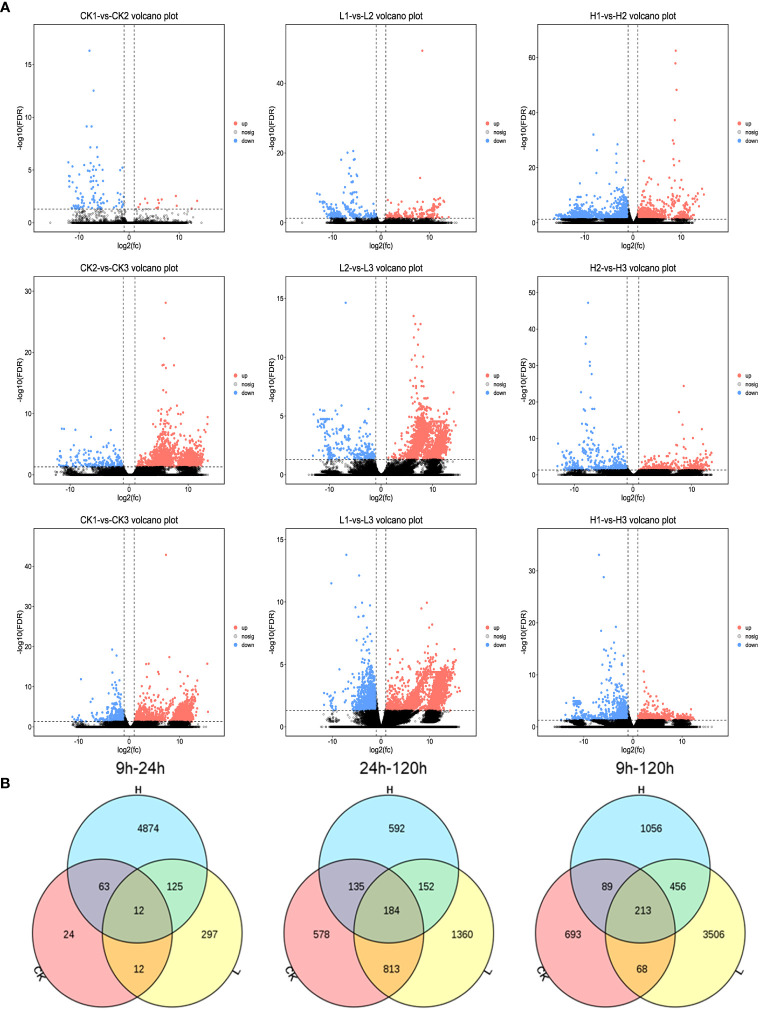
**(A)** Comparison of volcano plots with different periods at the same concentration. The number of DEGs in each tissue is presented and based on the significance shown in the volcano plots. The vertical dash lines in the volcano plots depict the two-fold differential expression cut-off (axis expressed as log2 values) and the horizontal dash lines shows the -log10(P-value). Dots in red (group_2 up-regulated relative to group_1) and blue (down-regulated) indicate differences in gene expression (FDR <0.05), while black dots show no differences. **(B)** Venn diagram of DEGs between various treatments.

Compared to CK, significantly fewer genes were upregulated in the high-concentration treatment group between 9h and 120h (**Figures 7C**, **8A**). In contrast, gene expression trends were similar between the low-concentration treatment group and CK across all treatment periods. However, the low-concentration treatment group showed a more significant degree of change in gene expression and a greater number of differential genes compared to the high-concentration treatment group (**Figure 8A**). Differential genes specific to the low-concentration treatment group were not significantly enriched in the KEGG pathway (**Figure 7F**). In the CK group, Ribosome, Glycolysis/Gluconeogenesis, and Oxidative phosphorylation were enriched between 9h and 120h, and were primarily associated with electron transport chain and hormone regulation (**Figure 7H**). We found fewer identical differential genes between the treatment groups and CK (**Figure 8B**), and *L. brownii* treated with different concentrations of 2,4-DTBP showed different growth patterns. No significant enrichment pathways were found in *L. brownii* subjected to low-concentration 2,4-DTBP treatment. However, GO annotation indicated that differential genes were involved in the response of *L. brownii* to acid, organic nitrogen compounds, and endogenous stimuli throughout the low-concentration treatment. High concentrations of 2,4-DTBP had a regulatory effect on the phytohormone and MAPK signaling pathways in *L. brownii.* Secondary metabolic pathways such as phenylpropane, flavonoids and Stilbenoid, diarylheptanoid, and gingerol biosynthesis were actively involved in the response to 2,4-DTBP.

### 3.6 2,4-DTBP got an impact on the oxidative phosphorylation and hormones of *L.brownii*

**Figure 9:**
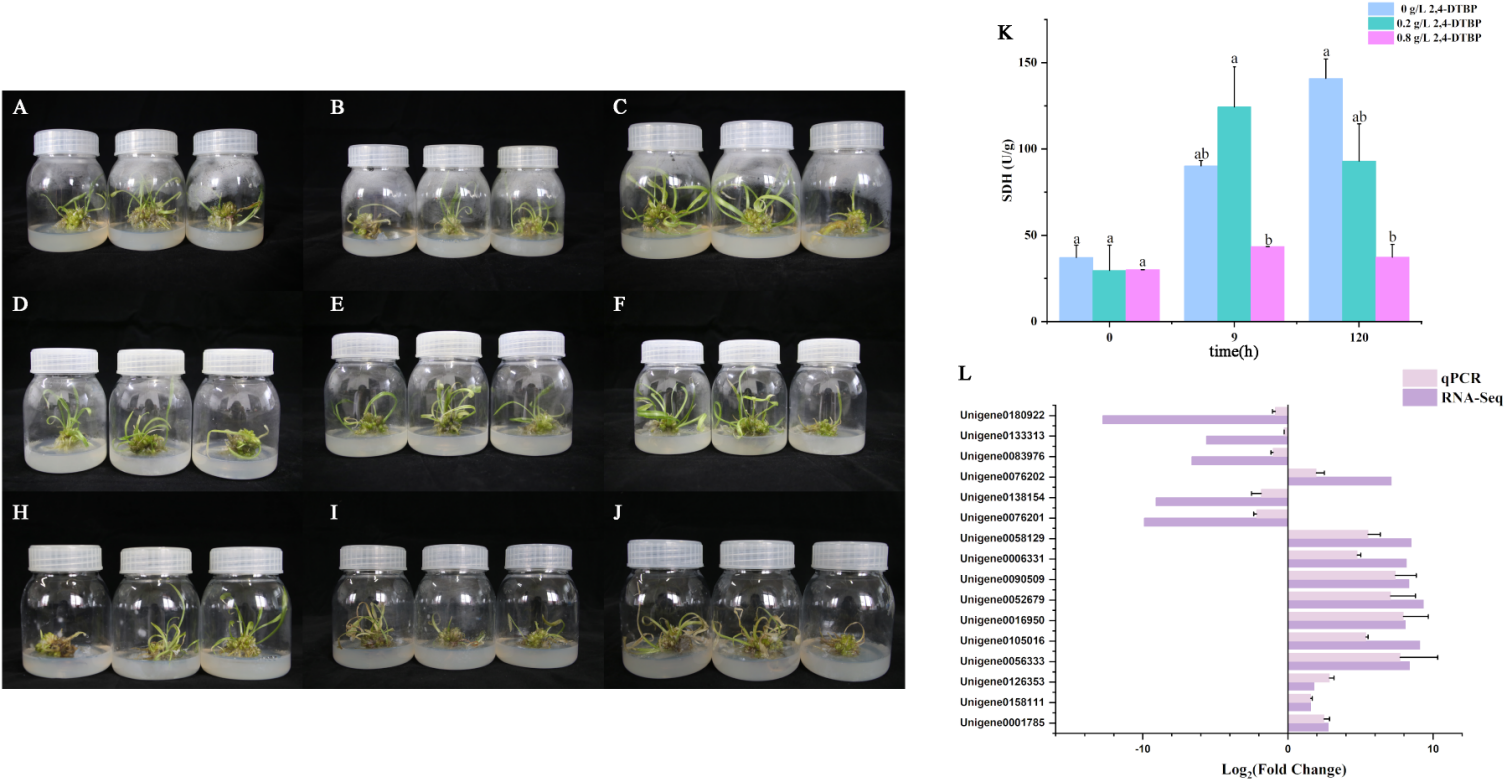
Tissue culture seedlings with consistent growth of L. brownii which treatment with 0 **(A–C)**, 0.2 **(D–F)** and 0.8 **(H–J)** g/L of 2,4-DTBP for 0, 9 and 120 hours. **(K)** The succinate dehydrogenase (SDH) activity of L. brownii. Values represent the mean + SEM (n=3). **(L)** QPCR result of DEGs in **Figures 6C, D** pertinent with oxidative phosphorylation and hormones pathways.

There was no significant difference observed in appearance between the control group and the treatment group after the first nine hours in touching with 2,4-DTBP (**Figures 9A–F**). The SDH activity was upregulated in the low concentration group with a short exposure (9h) to 2,4-DTBP compared to the control group(**Figure 9K**), and was significantly higher than that observed in the high concentration group. Leaf of the treatment group massive withered after 120 hours (**Figures 9H–J**), the SDH activity are decreased in two treatment groups than the control group. SDH activity of high concentration group remained suppressed throughout the experiment. DEGs in **Figures 6C, D** pertinent with oxidative phosphorylation and hormones pathways were selected for qPCR (**Figure 9L**), the results are the same as the transcriptome.

## 4 Discussion

Previous studies identified 2,4-DTBP as an autotoxic substance in *Lilium davidii* Duch. ex Elwes (Cui et al., 2022) and *Atractylodes macrocephala Koidz* (Zheng, 2018). Cui et al. (2022) found the rhizosphere of *Lilium davidii* exhibited reduced soil enzyme activity and a suppressed microbial community, and that 2 mmol/L of 2,4-DTBP increased the level of pathogenic fungi such as *Fusarium*. Recently, 2,4-DTBP has been frequently used as a weed control agent due to its broad-spectrum toxicity to plants (Chuah et al., 2015; Zain et al., 2021). A concentration of 0.8 g/L 2,4-DTBP had a higher inhibitory effect on root and stem length development and significantly inhibited germination of *Allium ascalonicum*.

2,4-DTBP is a phenol that affects the dynamic equilibrium of ROS in plant roots. Low concentrations of phenolic acids increase plant stress resistance (Mei et al., 2020; Chen et al., 2021; Migut et al., 2021). The accumulation of phenolic acids in the soil leads to soil acidification, where the cells of plant roots are the first to be affected. When phenolic acids enter cells with a higher pH, they dissociate to produce large amounts of hydrogen ions, resulting in a decrease in intracellular pH and depolarization of the intra-membrane environment. Intracellularly, phenolic acid autotoxic substances inhibit the activity of antioxidant enzymes, such as peroxidase (POD) and catalase (CAT), leading to an increase in ROS and lipid peroxidation (Zhang et al., 2018; Wang et al., 2019; Xiao et al., 2019; Marchiosi et al., 2020). Autotoxic substances caused a significant increase in the root cell ROS content of *Angelica Sinensis* (Xin et al., 2019). The determination of ROS in the root tips of treated seedlings indicated that phenolic acid-induced oxidative damage was an essential factor in the development of autotoxicity. Increasing ferulic acid concentration decreased the growth rate of *Oryza sativa* roots and increased ROS, calcium content, and lipoxygenase activity (Chi et al., 2013). Treatment with a low concentration of 2,4-DTBP inhibited POD and SOD in *L. brownii*, resulting in lipid peroxidation and an increase in MDA content. During high-concentration treatment, *L. brownii* may be affected by a combination of abiotic stresses such as osmotic stress, oxidative stress, and ionic stress. There was no significant difference between POD and SOD activity and CK for any of the time periods except 72h POD activity, which was significantly lower than CK.

Plant growth and development requires ATP which is produced by mitochondrial respiration. Complexes I-IV known as NADH ubiquinone oxidoreductase, succinate dehydrogenase, cytochrome *bc1* complex and Cytochrome c oxidase are responsible for the formation of the respiratory chain. Simultaneously, complex V is involved in electron transfer and the establishment of the electrochemical proton gradient across the inner mitochondrial membrane. The Results show that the SDH activity was decreased by long-term exposure to the medium containing the 2,4-DTBP. SDH related to regulates downstream defense and stress gene expression by being the source of mH_2_O_2_, and the defective SDH affecting mitochondrial electron activity (Gleason et al., 2011). Under Abiotic stress, ATP is consumed more as a fade-back mechanism. For example, ATP is used in large quantities to scavenge ROS and enhance the antioxidant capacity of plants under stress (Zhu et al., 2022). Excess reactive oxygen radicals disrupt the plant cell membrane structure and oxidize sulfhydryl groups of mitochondrial MPTP-associated proteins to disulfides, promoting MPTP opening. They also cause mitochondrial membrane peroxidation, which results in reduced membrane fluidity. The phenolic acid produced by continuous cropping causes an excessive accumulation of intracellular ROS by inhibiting the activity of enzymes such as SOD, POD, and CAT (Wang et al., 2019). Oxidative stress affects protein synthesis, and oxidants affect tRNA’s post-translational modification ability and stability (Shcherbik and Pestov, 2019). Wurtmann and Wolin (2009) found that oxidized RNA slows down protein synthesis, resulting in the production of aggregated and truncated peptides (Wurtmann and Wolin, 2009).

We isolated and screened the roots, stems, leaves, and bulbs of *Lilium brownii* and identified 2,4-DTBP as an autotoxic substance. The toxicity and enzymatic activity assay results of *L. brownii* following 2,4-DTBP treatment were used to screen three 2,4-DTBP concentrations and three time-point samplings for transcriptome sequencing.

Using the transcriptome data, we conducted a preliminary analysis of the effects of autotoxic substances on *L. brownii*. Different concentrations of 2,4-DTBP had varying effects on the growth and development of *L. brownii*. A response to external autotoxic effects was signaled by regulation of the MAPK-signaling and plant hormone signaling pathways, while secondary metabolic pathways, such as photosynthesis and phenylpropane synthesis and metabolism, were involved as downstream pathways for signaling responses to autotoxic substances. Our findings demonstrate hormonal changes at the transcriptional level in *L. brownii*. Endogenous plant hormones are affected by phenolic compounds (Desmedt et al., 2021). Following 2,4-DTBP treatment for 9h, the expression of phytohormone-related genes was upregulated in *L. brownii*. High BPA concentrations in soybean roots can inhibit root growth at the seedling stage by reducing the content of IAA, ZT, GA3, and ETH in roots and increasing the content of ABA (Li et al., 2018). This has also been observed in Arabidopsis (Bahmani et al., 2020). Hormones play a role in the response of plants to abiotic stresses (Zhou et al., 2018; Peng et al., 2021). For example, SA is related to the dynamic balance of plant ROS (La et al., 2019; Poór, 2020), and salt stress alters the content of plant hormones such as ABA and IAA (Wu et al., 2017). Additionally, ABA-promoted ROS in the mitochondria of Arabidopsis root tips is an essential retrograde signal that regulates root meristem activity by controlling auxin accumulation/signaling and PLT expression (Yang et al., 2014). Further studies are required to determine the specific changes in plant hormone content in response to autotoxic substances.

Plant hormone signaling, ROS, and the MAPK-signaling pathway are all involved in signaling activation and transmission in response to autotoxic substance stress. However, the mechanisms underlying these three processes needs further investigation. More research is required to confirm the specific effects of autotoxic substances on mitochondria and ribosomes and the role of each pathway in alleviating the onset of autotoxicity in *L. brownii*.

## Data availability statement

The datasets presented in this study can be found in online repositories. The names of the repository/repositories and accession number(s) can be found below: Bioproject accession number: PRJNA1056156.

## Author contributions

SZ: Conceptualization, Investigation, Writing – original draft, Writing – review & editing. CG: Conceptualization, Investigation, Writing – review & editing. LuS: Resources, Writing – review & editing. HJ: Resources, Writing – review & editing. X-EW: Resources, Writing – review & editing. LiS: Writing – review & editing, Supervision. XGL: Writing – review & editing. XLL: Writing – review & editing, Project administration, Supervision, Funding acquisition. JX: Project administration, Supervision, Writing – review & editing.

## Supplementary material

Supplementary Table 1 The list of the identified components in 4 tissues of *Lilium brownii.*

Supplementary Table 2 Quality statistics of Longya lily transcriptome sequencing.

Supplementary Table 3 9h-24h differential gene GO enrichment analysis(p. adjust<0.05).
