# Supplementary material for: Physiological and transcriptomic analyses provide preliminary insights into the autotoxicity of Lilium brownii
Source: Front Plant Sci. 2024 May 14;15:1330061. doi: 10.3389/fpls.2024.1330061 (PMC11130447; doi:10.3389/fpls.2024.1330061)
Supplement: Supplementary Table 1 — The list of the identified components in 4 tissues of Lilium brownii. [file Table_1.docx]

**Table S1** The list of the identified components in 4 tissues of *Lilium brownii*

|  | Root | Stem | Blub | Leaf |
| --- | --- | --- | --- | --- |
| 1 | Eicosane | 2,4-Di-tert-butylphenol | Hexadecane | 2-Bromo dodecane |
| 2 | Eugenol | 2-Ethyl-l-hexanol | 2-Methyltetracosane | Pentadecane |
| 3 | Heptane,2,2,3,3,5,6,6-heptamethyl- | 2-Isopropyl-5-methyl-1-heptanol | 2-Nonenal, (E)- | Acetic acid, 3-acetylcyclohexyl ester |
| 4 | Heptanoic acid | Acetic acid | Nonanal | Nonanal |
| 5 | Heptanoic acid,ethyl ester | Benzaldehyde, 2,4-dimethyl- | Heptanoic acid | 2-Furanone,2,5-dihydro-3,5-dimethyl |
| 6 | Hexanoic acid | cis-Thujopsene | 2-C14H26 | Ethanone,1-(1-cyclohexen-1-yl)- |
| 7 | Nonane,5-(2-methylpropyl) | Dodecane,4,6-dimethyl- | Decane,1,1'-oxybis- | p-Nitrophenyl hexanoate |
| 8 | Heptadecane | Dodecane,4,6-dimethyl-Hexadecane | Octanal | Dodecane, 2,5-dimethyl- |
| 9 | Hexanal | Dodecane,4-methyl- | Furan,2-pentyl- | Tetradecane |
| 10 | Pantolactone | Eicosane | 1-Octanol | Decane,4-methyl- |
| 11 | p-Cymene-2,5-diol | Heneicosane | Cyclodecane | Tridecane,6-methyl- |
| 12 | Tetradecane, I-iodo- | Heptadecane | Dodecane,4,6-dimethyl- | 2,5,5-Trimethyl-3-hexyn-2-ol |
| 13 | p-Mentha-1,5-dien-8-ol | Hexadecane | Undecane, 3,6-dimethyl- | Benzaldehyde |
| 14 | Thymol | Hexanal | Heptadecane | Octanoic acid |
| 15 | Pentadecane | Nonane, 4,5-dimethyl- | Tetradecane | Dodecane,2,6,11-trimethyl- |
| 16 | Phenol,2-methyl-5-(1-methylethyl)- | Nonane,5-(2-methylpropyl) | Hexadecane,2,6,11,15-tetramethyl- | Heptadecane,2,6,10,15-tetramethyl- |
| 17 | Phenylethyl Alcohol | Tetradecane | Nonane,5-(2-methylpropyl)- | Benzaldehyde,2,4-dimethyl- |
| 18 | Phenol, 4-ethyl-2-methoxy- | Undecane,3,6-dimethyl- | Decane, 3,8-dimethyl- | 3-Octen-2-one |
| 19 | Undecane,2,6-dimethyl- |  | 2,4-Di-tert-butylphenol | 7-Oxabicyclo[4.1.0]heptane, 1-methyl-4-(2-me |
| 20 | Phenol, 2,6-dimethoxy-4-(2-propenyl)- |  | 1-Nonanol | 5-Hepten-2-one,6-methyl- |
| 21 | p-Cymen-7-ol |  | Eicosane | 2-Octenal,(E)- |
| 22 | Octanoic acid |  | Decane,2,6,7-trimethyl- | 2(3H)-Furanone,dihydro-3-hydroxy-4,4-dimet |
| 23 | Nonanoic acid, ethyl ester |  | Decanal | Oxirane, decyl- |
| 24 | Hexadecane |  | 1-Undecene, 8-methyl- | 2-Nonenal,(E)- |
| 25 | He xadecane |  | 2-Octenal,(E)- | 3-Buten-2-one,4-(2,2,6-trimethyl-7-oxabicyck |
| 26 | Eucalyptol |  | 2-Heptenal,(E)- | 1-Octanol |
| 27 | Ethanone, 1-(6-methyl-7-oxabicyclo[4.1.0]hep |  | 2,6,10-Trimethyltridecane | Octanal |
| 28 | Dodecane,4-methyl- |  | Benzaldehyde,2,4-dimethyl- | 3-Butoxy-2,4-dimethyl-1-pentene |
| 29 | Dodecane,4,6-dimethyl- |  | 2,4-Nonadienal | 1-Cyclohexene-1-carboxaldehyde, 2,6,6-trimet |
| 30 | Dodecane,2,5-dimethyl- |  | Undecanal | Heneicosane |
| 31 | Dodecane |  | 3-Butoxy-2,4-dimethyl-1-pentene | Dodecane |
| 32 | Decanal |  | Sulfurous acid,2-pentyl tetradecyl ester | 5-Octen-2-one,6-ethyl- |
| 33 | Cyclobutanecarboxylic acid, 2-tetradecyl ester |  | n-Caproic acid vinyl ester | Dodecane, 4-methyl- |
| 34 | Butanoic acid, 3-methyl- |  | 2-Ethyl-1-hexanol | 2-Octanone |
| 35 | Benzenemethanol,.alpha.,.alpha.,4-trimethyl- |  | 1-Pentanol | 2(4H)-Benzofuranone,5,6,7,7a-tetrahydro-4,4 |
| 36 | Benzene,2-methoxy-4-methyl-1-(1-methylethy |  | 2(3H)-Furanone,dihydro-5-pentyl- | Eicosyl octyl ether |
| 37 | Benzene, 1,4-dimethoxy-2-methyl-5-isopropyl |  | Hexanoic acid | 2-Butenal,3-methyl- |
| 38 | Benzaldehyde,2,4-dimethyl- |  | Allyl 2-ethyl butyrate | 3,5-Octadien-2-one |
| 39 | Acetic acid |  | 5,9-Undecadien-2-one,6,10-dimethyl-,(E)- | 2,4-Heptadienal,(E,E)- |
| 40 | 3-tert-Butyl-4-hydroxyanisole |  | Hexanal | 2-Pentadecanone,6,10,14-trimethyl- |
| 41 | 3-Ethyl-3-methylheptane |  | 3,5-Octadien-2-ol | 2(3H)-Furanone, dihydro-3-hydroxy-4,4-dimet |
| 42 | 3,5-Dimethyldodecane |  | Heptanal | 3,5-Octadien-2-one,(E,E)- |
| 43 | 2-Oxabicyclo[2.2.2]octan-6-ol,1,3,3-trimethyl |  | Pantolactone | 2,5,5-Trimethyl-3-hexyn-2-ol |
| 44 | 2-Octen-1-ol,(E)- |  | 2(3H)-Furanone,5-ethyldihydro- | Decanal |
| 45 | 2-Nonenal,(E)- |  | Octanoic acid | 2,4-Di-tert-butylphenol |
| 46 | 2-Cyclohexen-1-ol,3-methyl-6-(1-methylethen |  | 1-Octen-3-o1 | 2-Heptanone, 3-propylidene- |
| 47 | 2-Cyclohexen-1-ol,1-methyl-4-(1-methylethen |  | Nonanoic acid | Ethanone, 1-(2-methyl-1-cyclopenten-1-yl)- |
| 48 | 2,6,10-Trimethyltridecane |  | Phenanthrene,7-ethenyl-1,2,3,4,4a,4b,5,6,7,9, | 1-Hepten-3-one |
| 49 | 2,4-Di-tert-butylphenol |  | n-Decanoic acid | 1,3-Cyclohexanediol,2-methyl-2-nitro-,mono; |
| 50 | 2,4-Diphenyl-4-methyl-1-pentene |  | 2(3H)-Furanone,dihydro-3-hydroxy-4,4-dimei | Hexanal |
| 51 | 2,2,4-Trimethyl-1,3-pentanediol diisobutyrate |  | Phenanthrene,7-ethenyl-1,2,3,4,4a,4b,5,6,7,8, | 5,9-Undecadien-2-one,6,10-dimethyl-,(Z)- |
| 52 | 1-Hexanol,2-ethyl- |  |  | Heptanoic acid |
| 53 | 1-Hexanol |  |  | 2-Undecanone,6,10-dimethyl- |
| 54 | 1H-2-Benzopyran-1-one,3,4-dihydro-8-hydro: |  |  | 3-Buten-2-one,4-(2,6,6-trimethyl-2-cyclohexe |
| 55 | 12-Heptadecyn-1-ol |  |  | Nonanoic acid |
| 56 | .alpha.-Terpineol |  |  | Hexanoic acid |
| 57 | (-)-cis-Isopiperitenol |  |  | Heptadecane |
| 58 |  |  |  | 4,4'-Biscyclohexanone, 2,2',6,6'-tetramethyl- |
| 59 |  |  |  | 2-Pentanol,3-chloro-2-methyl- |
| 60 |  |  |  | 2-Heptenal,(E)- |
| 61 |  |  |  | 2,5-Furandione,3,4-dimethyl- |
| 62 |  |  |  | 1-Undecene, 8-methyl- |
| 63 |  |  |  | 1-Octen-3-ol |
| 64 |  |  |  | 1H-Pyrrole-2,5-dione, 3-ethyl-4-methyl- |
| 65 |  |  |  | 1H-Pyrazole, 3-methyl- |
| 66 |  |  |  | 10-Methyl-8-tetradecen-l-ol acetate |
