# Supplementary material for: Physiological and transcriptomic analyses provide preliminary insights into the autotoxicity of Lilium brownii
Source: Front Plant Sci. 2024 May 14;15:1330061. doi: 10.3389/fpls.2024.1330061 (PMC11130447; doi:10.3389/fpls.2024.1330061)
Supplement: Supplementary Table 2 — Quality statistics of Longya lily transcriptome sequencing. [file Table_2.docx]

**Table S2** Quality statistics of Longya lily transcriptome sequencing

| sample | raw reads | clean reads(%) | poly A(%) | N(%) | Q20（%） | GC（%） |
| --- | --- | --- | --- | --- | --- | --- |
| CK1-1 | 37937896 | 37859158 (99.79%) | 0 (0.00%) | 0 (0.00%) | 5579379913 (98.67%) | 2813777587 (49.76%) |
| CK1-2 | 39143964 | 39055906 (99.78%) | 0 (0.00%) | 0 (0.00%) | 5751538356 (98.65%) | 2871158957 (49.25%) |
| CK1-3 | 43582180 | 43477898 (99.76%) | 0 (0.00%) | 0 (0.00%) | 6396303349 (98.54%) | 3230341468 (49.77%) |
| L1-1 | 46212498 | 46101112 (99.76%) | 0 (0.00%) | 0 (0.00%) | 6771953620 (98.44%) | 3419993436 (49.72%) |
| L1-2 | 40835130 | 40747554 (99.79%) | 0 (0.00%) | 0 (0.00%) | 5999009086 (98.63%) | 2990806806 (49.17%) |
| L1-3 | 48361166 | 48253052 (99.78%) | 0 (0.00%) | 0 (0.00%) | 7078334267 (98.64%) | 3550034049 (49.47%) |
| H1-1 | 38844264 | 38758240 (99.78%) | 0 (0.00%) | 0 (0.00%) | 5708371205 (98.62%) | 2819545511 (48.71%) |
| H1-2 | 40639050 | 40541156 (99.76%) | 0 (0.00%) | 0 (0.00%) | 5959349888 (98.67%) | 2984113170 (49.41%) |
| H1-3 | 57020874 | 56898060 (99.78%) | 0 (0.00%) | 0 (0.00%) | 8357596705 (98.55%) | 4170146563 (49.17%) |
| CK2-1 | 59629686 | 59503048 (99.79%) | 0 (0.00%) | 0 (0.00%) | 8759971709 (98.60%) | 4389619160 (49.41%) |
| CK2-2 | 52447060 | 52336358 (99.79%) | 0 (0.00%) | 0 (0.00%) | 7699088473 (98.63%) | 3838034749 (49.17%) |
| CK2-3 | 46362080 | 46263222 (99.79%) | 0 (0.00%) | 0 (0.00%) | 6807875833 (98.56%) | 3414773620 (49.44%) |
| L2-1 | 60987766 | 60839622 (99.76%) | 0 (0.00%) | 0 (0.00%) | 8944720218 (98.63%) | 4442845550 (48.99%) |
| L2-2 | 50524336 | 50412320 (99.78%) | 0 (0.00%) | 0 (0.00%) | 7414714873 (98.61%) | 3684707689 (49.00%) |
| L2-3 | 63057410 | 62917400 (99.78%) | 0 (0.00%) | 0 (0.00%) | 9274056817 (98.63%) | 4716528710 (50.16%) |
| H2-1 | 49242728 | 49114190 (99.74%) | 0 (0.00%) | 0 (0.00%) | 7228481673 (98.64%) | 3656038982 (49.89%) |
| H2-2 | 50869848 | 50735654 (99.74%) | 0 (0.00%) | 0 (0.00%) | 7461053232 (98.73%) | 3757184544 (49.72%) |
| H2-3 | 53421046 | 53265364 (99.71%) | 0 (0.00%) | 0 (0.00%) | 7817754511 (98.61%) | 3899592906 (49.19%) |
| CK3-1 | 52850476 | 52720256 (99.75%) | 0 (0.00%) | 0 (0.00%) | 7751511013 (98.66%) | 3802503223 (48.40%) |
| CK3-2 | 48177656 | 48045008 (99.72%) | 0 (0.00%) | 0 (0.00%) | 7056129508 (98.52%) | 3480219142 (48.59%) |
| CK3-3 | 58474958 | 58318422 (99.73%) | 0 (0.00%) | 0 (0.00%) | 8551738419 (98.40%) | 4246866228 (48.87%) |
| L3-1 | 52968698 | 52832352 (99.74%) | 0 (0.00%) | 0 (0.00%) | 7764024129 (98.60%) | 3870217997 (49.15%) |
| L3-2 | 59308450 | 59190920 (99.80%) | 0 (0.00%) | 0 (0.00%) | 8709044911 (98.70%) | 4405125170 (49.93%) |
| L3-3 | 58584086 | 58423392 (99.73%) | 0 (0.00%) | 0 (0.00%) | 8583543159 (98.60%) | 4221736134 (48.50%) |
| H3-1 | 53958608 | 53831652 (99.76%) | 0 (0.00%) | 0 (0.00%) | 7922398550 (98.70%) | 3894538170 (48.52%) |
| H3-2 | 51347468 | 51256576 (99.82%) | 0 (0.00%) | 0 (0.00%) | 7544174883 (98.64%) | 3730851817 (48.78%) |
| H3-3 | 56162262 | 56054462 (99.81%) | 0 (0.00%) | 0 (0.00%) | 8262887067 (98.62%) | 4173079389 (49.80%) |
