# Supplementary material for: Physiological and transcriptomic analyses provide preliminary insights into the autotoxicity of Lilium brownii
Source: Front Plant Sci. 2024 May 14;15:1330061. doi: 10.3389/fpls.2024.1330061 (PMC11130447; doi:10.3389/fpls.2024.1330061)
Supplement: Supplementary Table 3 — 9h-24h differential gene GO enrichment analysis(p. adjust<0.05). [file Table_3.docx]

**Table S3** 9h-24h differential gene GO enrichment analysis(p. adjust＜0.05)

| # | GO ID | description | p value | p. adjust |
| --- | --- | --- | --- | --- |
| 1 | GO:0006091 | generation of precursor metabolites and energy | 0 | 0.000002 |
| 2 | GO:0050896 | response to stimulus | 0 | 0.000007 |
| 3 | GO:0044699 | single-organism process | 0 | 0.000153 |
| 4 | GO:0006950 | response to stress | 0 | 0.000153 |
| 5 | GO:0009628 | response to abiotic stimulus | 0.000001 | 0.000153 |
| 6 | GO:0044711 | single-organism biosynthetic process | 0.000001 | 0.000153 |
| 7 | GO:0009411 | response to UV | 0.000001 | 0.000153 |
| 8 | GO:0042221 | response to chemical | 0.000002 | 0.000266 |
| 9 | GO:0022900 | electron transport chain | 0.000002 | 0.000281 |
| 10 | GO:0044550 | secondary metabolite biosynthetic process | 0.000003 | 0.000363 |
| 11 | GO:0015979 | photosynthesis | 0.000005 | 0.000584 |
| 12 | GO:0001101 | response to acid chemical | 0.000005 | 0.000584 |
| 13 | GO:0019684 | photosynthesis, light reaction | 0.000005 | 0.000584 |
| 14 | GO:0010033 | response to organic substance | 0.000013 | 0.00121 |
| 15 | GO:0009725 | response to hormone | 0.000013 | 0.00121 |
| 16 | GO:0009699 | phenylpropanoid biosynthetic process | 0.000014 | 0.00121 |
| 17 | GO:0001906 | cell killing | 0.00002 | 0.00166 |
| 18 | GO:0009605 | response to external stimulus | 0.000021 | 0.00166 |
| 19 | GO:0044283 | small molecule biosynthetic process | 0.000026 | 0.001953 |
| 20 | GO:0009719 | response to endogenous stimulus | 0.000035 | 0.002466 |
| 21 | GO:0009698 | phenylpropanoid metabolic process | 0.000037 | 0.002473 |
| 22 | GO:0006520 | cellular amino acid metabolic process | 0.000048 | 0.00307 |
| 23 | GO:1901700 | response to oxygen-containing compound | 0.000077 | 0.004737 |
| 24 | GO:0010817 | regulation of hormone levels | 0.000098 | 0.005587 |
| 25 | GO:0055114 | oxidation-reduction process | 0.000099 | 0.005587 |
| 26 | GO:0060918 | auxin transport | 0.000106 | 0.00575 |
| 27 | GO:0009767 | photosynthetic electron transport chain | 0.00011 | 0.005769 |
| 28 | GO:0034285 | response to disaccharide | 0.00012 | 0.006056 |
| 29 | GO:0009314 | response to radiation | 0.000138 | 0.006712 |
| 30 | GO:0043476 | pigment accumulation | 0.000156 | 0.006712 |
| 31 | GO:0043478 | pigment accumulation in response to UV light | 0.000156 | 0.006712 |
| 32 | GO:0043479 | pigment accumulation in tissues in response to UV light | 0.000156 | 0.006712 |
| 33 | GO:0043480 | pigment accumulation in tissues | 0.000156 | 0.006712 |
| 34 | GO:0043473 | pigmentation | 0.000169 | 0.006834 |
| 35 | GO:0065008 | regulation of biological quality | 0.000171 | 0.006834 |
| 36 | GO:0009416 | response to light stimulus | 0.000174 | 0.006834 |
| 37 | GO:0044710 | single-organism metabolic process | 0.000202 | 0.007626 |
| 38 | GO:0009914 | hormone transport | 0.000205 | 0.007626 |
| 39 | GO:0042546 | cell wall biogenesis | 0.000231 | 0.008071 |
| 40 | GO:0043436 | oxoacid metabolic process | 0.000239 | 0.008071 |
| 41 | GO:0009617 | response to bacterium | 0.000242 | 0.008071 |
| 42 | GO:0006082 | organic acid metabolic process | 0.000242 | 0.008071 |
| 43 | GO:0008643 | carbohydrate transport | 0.000245 | 0.008071 |
| 44 | GO:0010035 | response to inorganic substance | 0.000276 | 0.008881 |
| 45 | GO:0006970 | response to osmotic stress | 0.0003 | 0.009435 |
| 46 | GO:0044763 | single-organism cellular process | 0.000316 | 0.009628 |
| 47 | GO:0006873 | cellular ion homeostasis | 0.00032 | 0.009628 |
| 48 | GO:0006732 | coenzyme metabolic process | 0.000337 | 0.009828 |
| 49 | GO:0055082 | cellular chemical homeostasis | 0.00034 | 0.009828 |
| 50 | GO:0016053 | organic acid biosynthetic process | 0.00041 | 0.011374 |
| 51 | GO:0046394 | carboxylic acid biosynthetic process | 0.00041 | 0.011374 |
| 52 | GO:0019752 | carboxylic acid metabolic process | 0.000478 | 0.013012 |
| 53 | GO:0022904 | respiratory electron transport chain | 0.000625 | 0.016514 |
| 54 | GO:0051186 | cofactor metabolic process | 0.00063 | 0.016514 |
| 55 | GO:0006558 | L-phenylalanine metabolic process | 0.000722 | 0.017632 |
| 56 | GO:1902221 | erythrose 4-phosphate/phosphoenolpyruvate family amino acid metabolic process | 0.000722 | 0.017632 |
| 57 | GO:0032535 | regulation of cellular component size | 0.000722 | 0.017632 |
| 58 | GO:0090066 | regulation of anatomical structure size | 0.000722 | 0.017632 |
| 59 | GO:0019725 | cellular homeostasis | 0.000754 | 0.018088 |
| 60 | GO:1901605 | alpha-amino acid metabolic process | 0.000774 | 0.018258 |
| 61 | GO:0008610 | lipid biosynthetic process | 0.000823 | 0.019097 |
| 62 | GO:0009743 | response to carbohydrate | 0.000866 | 0.019782 |
| 63 | GO:0008652 | cellular amino acid biosynthetic process | 0.000985 | 0.021855 |
| 64 | GO:0044281 | small molecule metabolic process | 0.000988 | 0.021855 |
| 65 | GO:0005975 | carbohydrate metabolic process | 0.001327 | 0.028898 |
| 66 | GO:0015980 | energy derivation by oxidation of organic compounds | 0.00136 | 0.029174 |
| 67 | GO:0050801 | ion homeostasis | 0.001398 | 0.029551 |
| 68 | GO:0019748 | secondary metabolic process | 0.001442 | 0.029779 |
| 69 | GO:0006790 | sulfur compound metabolic process | 0.001451 | 0.029779 |
| 70 | GO:0044272 | sulfur compound biosynthetic process | 0.001567 | 0.031692 |
| 71 | GO:0000103 | sulfate assimilation | 0.001818 | 0.036008 |
| 72 | GO:0010109 | regulation of photosynthesis | 0.001831 | 0.036008 |
| 73 | GO:1901564 | organonitrogen compound metabolic process | 0.001992 | 0.038635 |
| 74 | GO:0032501 | multicellular organismal process | 0.002448 | 0.046319 |
| 75 | GO:0006733 | oxidoreduction coenzyme metabolic process | 0.002453 | 0.046319 |
| 76 | GO:0051188 | cofactor biosynthetic process | 0.002505 | 0.046678 |
| 77 | GO:0031399 | regulation of protein modification process | 0.002711 | 0.049852 |
